# Risk of COVID‐19 infection in adult patients with atopic eczema and psoriasis: a single‐centre cross‐sectional study

**DOI:** 10.1111/bjd.20062

**Published:** 2021-08-01

**Authors:** Z.Z.N. Yiu, G. Harding‐Oredugba, C.E.M. Griffiths, R.B. Warren, E. McMullen, H.J.A. Hunter

**Affiliations:** Centre for Dermatology Research Salford Royal NHS Foundation Trust The University of Manchester Manchester Academic Health Science Centre National Institute for Health Research (NIHR) Manchester Biomedical Research Centre ManchesterUK; Business Information Department Salford Royal NHS Foundation Trust Manchester Academic Health Science Centre Manchester UK; Centre for Dermatology Research Salford Royal NHS Foundation Trust The University of Manchester Manchester Academic Health Science Centre National Institute for Health Research (NIHR) Manchester Biomedical Research Centre ManchesterUK; Centre for Dermatology Research Salford Royal NHS Foundation Trust The University of Manchester Manchester Academic Health Science Centre National Institute for Health Research (NIHR) Manchester Biomedical Research Centre ManchesterUK; Centre for Dermatology Research Salford Royal NHS Foundation Trust The University of Manchester Manchester Academic Health Science Centre National Institute for Health Research (NIHR) Manchester Biomedical Research Centre ManchesterUK; Centre for Dermatology Research Salford Royal NHS Foundation Trust The University of Manchester Manchester Academic Health Science Centre National Institute for Health Research (NIHR) Manchester Biomedical Research Centre ManchesterUK

Dear Editor, Many studies have investigated risk factors for poor outcomes following COVID‐19. These studies are important for planning targeted prevention and/or intervention. A UK cohort study found that a composite variable of autoimmune diseases, representing rheumatoid arthritis, lupus or psoriasis, was associated with an increased risk of death owing to COVID‐19 [hazard ratio 1·19, 95% confidence interval (CI) 1·11–1·27].^1^ In contrast, a case series of patients with COVID‐19 in two US centres found that atopic eczema (AE) was associated with a reduction in the risk of hospitalization in patients with COVID‐19 [odds ratio (OR) 0·51, 95% CI 0·25–0·90].^2^ Most published studies investigated the risk of infection with SARS‐CoV‐2 and poor COVID‐19 outcomes associated with oral or biological treatment for psoriasis/eczema rather than for the condition itself.^3^ Our aim was to investigate the risk of COVID‐19 infection associated with having psoriasis or AE in a UK tertiary dermatology centre [Salford Royal NHS Foundation Trust (SRFT), Manchester, UK].

We performed a cross‐sectional study using data extracted from the SRFT electronic patient records (EPRs) of inpatient and outpatient visits. SRFT hosts one of the largest UK dermatology departments; a tertiary psoriasis clinic; and one of the few inpatient dermatology wards in the country. We included all patients aged ≥ 18 years who had one or more visits to the SRFT dermatology service between June 2018 and February 2021. Our exposure of interest was an inpatient or outpatient diagnosis of psoriasis or AE. We excluded all individuals who did not reside in Salford as they were unlikely to have presented to SRFT for COVID‐19 testing.

Clinical diagnoses were coded using the International Classification of Diseases, 10th Revision codes for inpatient admissions. Outpatient diagnoses [including comorbidities of hypertension, chronic obstructive pulmonary disease and diabetes mellitus (DM)] were extracted from letters. Data on immunosuppressive treatments were extracted from letters when vulnerable adults were identified for targeted protection measures (‘shielding’), during the COVID‐19 pandemic, on the advice of the UK government in March 2020.^4^ The most up‐to‐date values for age, body mass index (BMI), ethnicity and sex were extracted from the EPR. Our outcome of interest was patients who had a positive polymerase chain reaction SARS‐CoV2 swab test. We also identified individuals who were admitted to hospital for management of COVID‐19. The descriptive data were summarized by median and interquartile range for continuous data, and by number and percentage for dichotomous/categorical variables. We fitted logistic regression models with COVID‐19 diagnosis as the outcome and psoriasis or AE as the exposure, additionally adjusting for potential confounders (median‐centred age, sex, ethnicity, BMI) and potential mediators between the exposure and the outcome (hypertension and DM) in separate complete‐case and multiply imputed (MI) (20 sets) models.

Information for 56 835 patients was extracted; 13 162 patients were eligible for inclusion. There were 1427 (10·8%) patients with psoriasis and 624 (4·7%) with AE. In total, 176 (1·3%) of the eligible patients had COVID‐19, 38 (21·6%) of whom were hospitalized [two with psoriasis (who recovered), none with AE]. Baseline demographic data are presented in Table 1. We did not find a statistically significant elevated risk for infection with COVID‐19 in patients with psoriasis [unadjusted OR 0·60 (95% CI 0·33–1·08), complete‐case adjusted OR 0·98 (95% CI 0·46–2·08), MI adjusted OR 0·50 (95% CI 0·28–0·92)] or AE [unadjusted OR 0·71 (95% CI 0·31–1·60), complete‐case adjusted OR 0·60 (95% CI 0·22–1·64), MI adjusted OR 0·67 (95% CI 0·29–1·53)].

**Table 1:** Characteristics of study population by COVID‐19 infection status

Patient characteristic	Individuals with no history of COVID‐19 infection (*N* = 12 986)	Individuals with history of COVID‐19 infection (*N* = 176)
Age, years	55·0 (36·0–71·0)	75·0 (59·0–83·0)
Sex		
Male	5464 (42·1)	88 (50·0)
Female	7522 (57·9)	88 (50·0)
Body mass index	27·4 (24·0–31·6)	28·3 (24·9–32·9)
Missing	7609 (57·2)	53 (30·1)
Ethnicity		
White	12 157 (93·6)	173 (98·3)
Afro‐Caribbean	69 (0·5)	0 (0·0)
South Asian	154 (1·2)	3 (1·7)
Other Asian	144 (1·1)	0 (0·0)
Mixed	65 (0·5)	0 (0·0)
Other ethnic groups	159 (1·2)	0 (0·0)
Not recorded	238 (1·8)	0 (0·0)
Disease exposures		
Psoriasis	1415 (10·9)	12 (6·8)
Atopic eczema	618 (4·8)	6 (3·4)
Hypertension	100 (0·8)	6 (3·4)
Chronic obstructive pulmonary disease	16 (0·1)	0 (0·0)
Diabetes	63 (0·5)	1 (0·6)
Systemic treatment history		
Tumour necrosis factor inhibitor	68 (0·5)	0 (0·0)
Interleukin 17/23 inhibitor	41 (0·3)	0 (0·0)
Prednisolone	20 (0·2)	0 (0·0)
Dupilumab	18 (0·1)	0 (0·0)
Admission owing to COVID‐19	0 (0·0)	38 (21·6)

Continuous data are presented as median and interquartile range; dichotomous/categorical variables are presented as *n* (%).

A diagnosis of psoriasis or AE was not associated with an increase in the risk of testing positive for COVID‐19 compared with other patients attending the dermatology department for other conditions such as skin cancer. One of the strengths of this study is the inclusion of a generalizable population of patients with psoriasis and AE, regardless of treatment. The limitations of this study include potential misclassification of confounders (owing to missing information from letters) and outcome (community COVID‐19 test results were not available), lack of adjustment for potential confounders such as smoking, and effect estimate imprecision. Additionally, patients with inflammatory skin diseases may practice stricter shielding measures, which could explain the halving in risk for psoriasis in the MI adjusted analysis. It has been shown that people with psoriasis receiving targeted biological and systemic therapies are likely to follow the most stringent risk‐mitigating behaviours.^5^ In conclusion, psoriasis and AE were not associated with an increased risk of testing positive for COVID‐19. On this evidence, it appears that psoriasis and AE should not be considered as risk factors for contracting COVID‐19. Further research in larger cohorts with representative denominators is needed to confirm this finding.

## Author Contribution

**Zenas Zee Ngai Yiu:** Conceptualization (lead); Data curation (lead); Formal analysis (lead); Methodology (lead); Writing‐original draft (lead); Writing‐review & editing (lead). **Grace Harding‐Oredugba:** Data curation (equal); Writing‐review & editing (supporting). **Christopher Ernest, Maitland Griffiths:** Supervision (equal); Writing‐review & editing (equal). **Richard B Warren:** Supervision (equal); Writing‐review & editing (equal). **Emma McMullen:** Conceptualization (equal); Resources (equal); Supervision (equal); Writing‐review & editing (equal). **Hamish J.A. Hunter:** Conceptualization (equal); Supervision (lead); Writing‐review & editing (equal).
